# Second primary malignancies in cervical cancer and endometrial cancer survivors: a population-based analysis

**DOI:** 10.18632/aging.204047

**Published:** 2022-05-04

**Authors:** Kejie Huang, Lijuan Xu, Mingfang Jia, Wenmin Liu, Shijie Wang, Jianglong Han, Yanbo Li, Qibin Song, Zhenming Fu

**Affiliations:** 1 Cancer Center, Renmin Hospital of Wuhan University, Wuhan 430060, China; 2 Department of Health Management, Renmin Hospital of Wuhan University, Wuhan 430060, China; 3 Department of Gynecology and Obstetrics, Renmin Hospital of Wuhan University, Wuhan 430060, China

**Keywords:** second primary malignancies, radiotherapy, cervical cancer, endometrial cancer, Surveillance, Epidemiology, and End Results (SEER) registry

## Abstract

Background: We evaluated the relative attribution and interactions of treatment and patient-related risk factors for second primary malignancies (SPMs) in cervical and endometrial cancer survivors.

Methods: Stage I–III cervical and endometrial cancer survivors’ data from the Surveillance, Epidemiology, and End Results (SEER) registry between January 1988 and December 2015 were analyzed. The standardized incidence ratio (SIR), excess absolute risk (EAR), and corresponding 95% confidence interval (95% CI) values were calculated. Analyses were classified based on proxies of human papillomavirus (HPV), smoking, hormone, and radiotherapy (RT) status. Additive and multiplicative interactions were assessed.

Results: Cervical cancer survivors had a higher risk for developing potentially HPV and smoking-related SPMs, especially in the RT group (SIR_HPV_ = 3.7, 95% CI: 2.9–4.6; SIR_smoking_ = 3.2, 95% CI: 2.8–3.6). Second vaginal cancer patients had the highest SIR (23.8, 95% CI: 14.9–36.0). There were strong synergistic interactions between RT and the proxy of smoking (*P*_interaction_ < 0.001), accounting for 36% of potentially smoking-related SPMs in cervical cancer survivors.

Conclusions: RT, HPV, and smoking promote SPMs in cervical cancer to different extents. The SPM burden in cervical cancer survivors could be mostly attributed to smoking and RT and their interactions.

## INTRODUCTION

Cervical and endometrial cancers are common in women. After treatment, the 5-year survival rate of cervical cancer patients is 66% and reaches 81% in endometrial cancer patients [1, 2]. More than 283 and 807 thousand Americans with a history of cervical and endometrial cancer were alive on January 1, 2019. There are an estimated 288,710 cervical cancer survivors and an estimated 1,023,290 endometrial cancer survivors by January 1, 2030 in the United States [2]. Radiotherapy (RT) prolongs the survival rate of patients with locally advanced cervical and endometrial cancers [3–5]. The prolonged survival means follow-up evaluation of these patients is important not only for disease control but also for early detection of late events such as second primary malignancies (SPMs).

SPMs develop after the initial primary malignancy [6]. The risk of SPMs is associated with various factors [7], including continuous exposure to lifestyle factors (e.g., smoking), genetic factors, etiological factors (e.g., human papillomavirus [HPV]), and treatments such as radiation therapy. Moreover, potential complex interactions between these risk factors might cause SPMs [8]. HPV infection is the primary cause of cervical cancer [9]. It still affects cervical cancer patients even after successful treatment [10, 11]. Furthermore, the estimated smoking prevalence among cervical cancer survivors exceeds 40%, whereas the smoking rate in the general population of US women is only 18% [9]. Continuous exposure to these risk factors puts cervical cancer survivors at high risk for SPMs. Endometrial and cervical cancers are anatomically similar but etiologically different from each other. Endometrial cancer is associated with hormone-related factors [12]. Similarly, hormone-related risk factors and long-term effects of treatment can also cause SPMs in endometrial cancer survivors. Radiotherapies for both cancers are similar. However, no significant association has been found between endometrial cancer and smoking or HPV [13].

Counseling on SPM risk and seeking active measures to minimize the risk becomes pertinent as cervical and endometrial cancer survivors increase in prevalence [2, 11], though the impact of risk factors on SPM development in these two cancers remains unclear. Previous studies usually focused on the role of individual risk factors and ignored the contribution of their potential interactions in SPM risk [14]. Using data from the Surveillance, Epidemiology, and End Results (SEER) registry, we aimed to examine the determinants and their interactions in SPM risk in survivors of cervical and endometrial cancer, taking advantage of their similar treatments and anatomy, as well as the different etiology of endometrial cancer.

## METHODS

### Study population and data sources

**Figure 1:**
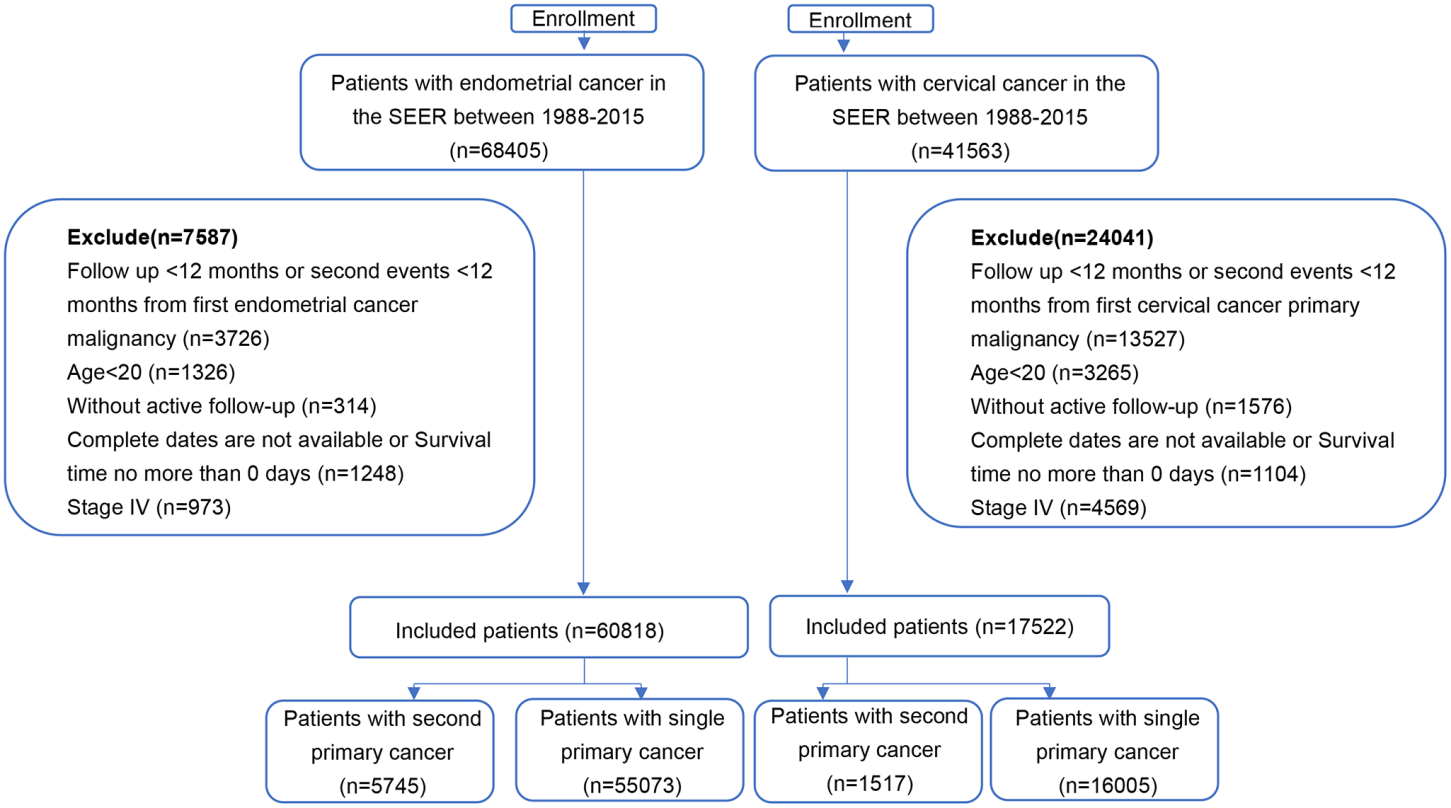
**The flowchart of study population selection.** Abbreviation: SEER: Surveillance, Epidemiology, and End Results.

The SEER database covers approximately 97% of all cancer incidences in registry areas within the United States. The database records basic demographics and some clinical characteristics [15]. SEER*stat software (version 8.3.8) was used to select patients. Eligible participants diagnosed with index primary cervical or endometrial cancer between January 1988 and December 2015 were identified (Figure 1). Patients over 20 years old with at least 12 months of follow-up were included. SPMs were defined as a new primary cancer occurring at least 12 months after an index cancer [16]. A one-year latency after the diagnosis of initial cancer was required to exclude recurrence or metastases of the first neoplasm [16]. Stage IV patients were also excluded to avoid metastases of primary cancer. Additionally, patients diagnosed via only autopsy or death certificate were excluded, as follow-up information was not available. All patients had complete data such as year of diagnosis, age, race, marital status, disease stage, RT (yes or no/unknown), chemotherapy (yes or no/unknown), and surgery (yes or no/unknown). The study ended at the diagnosis date of SPMs, the death of patients, or the end date of the study (December 31, 2015), whichever came first.

Secondary cervical or endometrial cancers were not included as eligible sites of SPMs to avoid the inclusion of relapses in the analyses [17]. Proxies of risk factors were used. SPMs were dichotomized into potentially HPV-related or non-HPV-related SPMs, potentially smoking-related or non-smoking-related SPMs, and potentially hormone-related SPMs or non-hormone-related SPMs since smoking and HPV information was not available in the SEER data. Dichotomous variables were used as substituents for HPV infection, smoking, and hormone status. Oropharyngeal cancers (tongue, tonsil, oropharynx) [18] and cancers of the vagina, vulva, anus, and rectum were usually associated with HPV infection and were used as proxies of HPV-related SPMs. Cancers of the esophagus, lung, bronchus, and bladder were classified as potentially smoking-related SPMs [18, 19]. In addition, female breast and ovarian cancers were grouped as potentially hormone-related SPMs to compare cervical and endometrial cancer survivors [20]. All cancers were histologically defined by ICD-O-3 codes in the SEER.

### Statistical analysis

Chi-square tests were used to compare the distribution of demographic characteristics. Univariate and multivariable analyses were performed using a Cox proportional hazards model to assess the hazard ratio (HR), with a 95% confidence interval (CI). Significant variables in univariate analysis were selected for multivariable analysis. Multiplicative interactions between risk factors were evaluated by including interaction terms in the final multivariable models of Cox regression analyses. Additive interactions were assessed using the calculation defined by Rothman and Anderson et al. through the following three measures of biological interaction: the relative excess risk due to interaction (RERI), the attribution proportion due to interaction (AP), and the synergy index (S) [21]. Several combinations, such as positive multiplicative positive additive, no multiplicative positive additive, negative multiplicative positive additive, and negative multiplicative negative additive interaction, were used to account for the possible complex interactions [22].

The standardized incidence ratio (SIR) and excess absolute risk (EAR) were estimated by comparing with age-matched female in the general population [9] to evaluate the risk of developing the SPMs in primary cervical and endometrial cancer survivors. Specifically, the SIR with 95% CI was defined as the ratio of observed [O]/expected [E] number of patients diagnosed. The expected number of patients was based on the cancer incidence rates in the United States standard population, adjusted for several variables like person-years of follow-up, age, race, and sex to ensure the most appropriate comparison [6, 23]. EAR (per 10,000 person-years) was calculated as the observed number of second cancers minus the expected number of second cancers/person-years at risk and then multiplied by 10,000 [24]. Sensitivity analyses were conducted using latency years, stage, age, and implementation of hysterectomy and/or ovariectomy of index primary cancer to test the robustness of the results. We also conducted sensitivity analyses by classifying mouth, throat, larynx, esophagus, lung, liver, stomach, pancreas, bladder, kidney, prostate, colon, and rectum as potentially smoking-related SPMs. A *P* value ≤ 0.05 (2-sided) was considered statistically significant. To address the issue of multiple comparison, adjusted methods are usually used to evaluate the significance of a test statistic. We thus applied Bonferroni correction to the analysis, because it is a commonly used conservative method for taking the multiplicity into account. After the Bonferroni correction, a *P* value ≤ 0.017 was considered statistically significant in the multiple comparisons. All analyses were conducted using SPSS 25 (IBM Corp, Armonk, NY, USA) and SEER*stat software (version 8.3.8).

### Ethics statement

Patient consents were not required because this is a retrospective database research in nature. Institutional Review Board approval was not required.

### Data availability

Data are accessible in the SEER database and available on request.

## RESULTS

Table 1 shows the characteristic distributions of the patients by RT status. Cervical cancer patients with no RT tended to be younger and white, have localized stage, have received surgery, and not have received chemotherapy. Endometrial cancer patients with the regional stage who received chemotherapy were more likely to receive RT. Most cervical cancer patients were between 35 and 50 years old, while endometrial cancer patients were over 50 years old. The number of cervical cancer patients who received RT was similar to that of patients who did not. However, the number of endometrial cancer patients who did not receive RT was nearly triple the number of those who did. There were substantial differences between groups with RT and no RT (*P* < 0.001).

**Table 1:** Characteristics of the index cervical cancer and endometrial cancer survivors from the Surveillance, Epidemiology, and End Results (SEER) database, 1988–2015.

**Baseline characteristic**		**Total, *n***	**Cervical cancer, RT, *n* (%)**	**Cervical cancer, no RT, *n* (%)**	^*^ *P*	**Total, *n***	**Endometrial cancer, RT, *n* (%)**	**Endometrial cancer, no RT, *n* (%)**	^*^ *P*
Year of diagnosis	1988–2004	12125	5299 (43.70)	6826 (56.30)	<0.001	30463	8285 (27.20)	22178 (72.80)	0.024
	2005–2015	5397	2745 (50.86)	2652 (49.14)		30355	8009 (26.38)	22346 (73.62)	
Age group	<35	3694	831 (22.50)	2863 (77.50)	<0.001	908	100 (11.01)	808 (88.99)	<0.001
	35-49	7185	2979 (41.46)	4206 (58.54)		7951	1485 (18.68)	6466 (81.32)	
	50-64	3986	2445 (61.34)	1541 (38.66)		27163	6812 (25.08)	20351 (74.92)	
	≥65	2657	1789 (67.33)	868 (32.67)		24796	7897 (31.85)	16899 (68.15)	
Race	White	12689	5556 (43.79)	7133 (56.21)	<0.001	50818	13819 (27.19)	36999 (72.81)	<0.001
	Black	2629	1415 (53.82)	1214 (46.18)		4102	1243 (30.30)	2859 (69.70)	
	Other	2204	1073 (48.68)	1131 (51.32)		5898	1232 (20.89)	4666 (79.11)	
Stage of disease	Local	10321	2273 (22.02)	8048 (77.98)	<0.001	48964	9886 (20.19)	39078 (79.81)	<0.001
	Regional	6137	5500 (89.62)	637 (10.38)		10237	6159 (60.16)	4078 (39.84)	
	Unknown	1064	271 (25.47)	793 (74.53)		1617	249 (15.40)	1368 (84.60)	
Marital status	No	3988	1730 (43.38)	2258 (56.62)	<0.001	9515	2431 (25.55)	7084 (74.45)	0.003
	Yes	13534	6314 (46.65)	7220 (53.35)		51303	13863 (27.02)	37440 (72.98)	
Surgery^†^	No	5364	4442 (82.81)	922 (17.19)	<0.001	2759	742 (26.89)	2017 (73.11)	0.901
	Yes	12158	3602 (29.63)	8556 (70.37)		58059	15552 (26.79)	42507 (73.21)	
Chemotherapy	No	13007	3779 (29.05)	9228 (70.95)	<0.001	55669	13091 (23.52)	42578 (76.48)	<0.001
	Yes	4515	4265 (94.46)	250 (5.54)		5149	3203 (62.21)	1946 (37.79)	

Abbreviations: RT: received radiotherapy; no RT: not received radiotherapy; *n*: number of cases. ^*^P values were derived from Chi-square analyses. ^†^Surgery of primary sites: a surgical procedure that removes and/or destroys the tissue of the primary sites.

As shown in Table 2, cervical cancer patients of older age, black race, regional stage, and RT tended to have SPMs, cervical cancer survivors of young age, black race, local stage and not received RT seem to have low risk of SPMs. Similarly, endometrial cancer patients of older age, white race, regional stage, and RT were more likely to have SPMs. The median age at diagnosis of primary cervical cancer was 53 years old; the median age at the time of SPMs diagnosis was 61 years old; the median latency from primary cervical cancer to SPMs was 90 months. The median at diagnosis of primary endometrial cancer was 64 years old; the median at the time of SPMs diagnosis was 71 years old; the median from primary endometrial cancer to SPMs was 72 months. In addition, radiotherapy increased SPM risk in both cervical (1.4, 95% CI: 1.2–1.6) and endometrial cancer survivors (1.2, 95% CI: 1.1–1.3). However, chemotherapy did not significantly affect the risk of SPMs in survivors of either cancer after multivariate adjustment (*P* > 0.05).

**Table 2:** Selected risk factors for the second primary malignancies (SPMs) from cervical cancer and endometrial cancer survivors, SEER, 1988–2015.

**Risk factors**		**Cervical cancer**				**Endometrial cancer**			
		**Total, *n***	**only single primary cancer, *n*/SPMs, *n***	^*^ *P*	**HR^‡^ (95% CI)**	**Total, *n***	**only single primary cancer, *n*/SPMs, *n***	^*^ *P*	**HR^‡^ (95% CI)**
Year of diagnosis	1988–2004	12125	10802/1323	<0.001	Reference	30463	26214/4249	<0.001	Reference
	2005–2015	5397	5203/194		0.97 (0.82–1.14)	30355	28859/1496		1.0 (0.9–1.1)
Age group	<35	3694	3552/142	<0.001	Reference	908	867/41	<0.001	Reference
	35–49	7185	6661/524		2.2 (1.8–2.6)	7951	7418/533		1.4 (1.0–1.9)
	50–64	3986	3499/487		4.3 (3.5–5.2)	27163	24821/2342		2.0 (1.5–2.9)
	≥65	2657	2293/364		7.0 (5.7–8.7)	24796	21967/2829		3.4 (2.5–4.6)
Race	White	12689	11597/1092	0.001	Reference	50818	45843/4975	<0.001	Reference
	Black	2629	2362/267		1.2 (1.0–1.4)	4102	3781/321		1.1 (1.0–1.3)
	Other	2204	2046/158		0.71 (0.60–0.84)	5898	5449/449		0.95 (0.86–1.05)
Stage of disease	Local	10321	9478/843	0.010	Reference	48964	44224/4740	<0.001	Reference
	Regional	6137	5573/564		1.1 (1.0–1.3)	10237	9373/864		1.1 (1.0–1.2)
	Unknown	1064	954/110		1.3 (1.0–1.6)	1617	1476/141		1.1 (0.9–1.3)
Marital status	No	3988	3732/256	<0.001	Reference	9515	8792/723	<0.001	Reference
	Yes	13534	12273/1261		1.0 (0.9–1.2)	51303	46281/5022		1.0 (0.9–1.1)
Chemotherapy	No	13007	11818/1189	<0.001	Reference	55669	50225/5444	<0.001	Reference
	Yes	4515	4187/328		1.1 (0.9–1.2)	5149	4848/301		1.0 (0.9–1.1)
Surgery ^†^	No	5364	4850/514	0.004	Reference	2759	2561/198	<0.001	Reference
	Yes	12158	11155/1003		0.82 (0.72–0.94)	58059	52512/5547		0.87 (0.74–1.03)
Radiotherapy	No	9478	8752/726	<0.001	Reference	44524	40455/4069	<0.001	Reference
	Yes	8044	7253/791		1.4 (1.2–1.6)	16294	14618/1676		1.2 (1.1–1.3)

Abbreviations: SEER, Surveillance, Epidemiology, and End Results; *n*: number of cases; HR: hazard ratio; CI: confidence interval; “-”: not available. ^*^P values were derived from Chi-square analyses. ^†^Surgery of primary sites: a surgical procedure that removes and/or destroys the tissue of the primary sites. ^‡^Hazard ratios were derived from multivariate analysis of Cox proportional hazards model. All the variables significant in the univariate analysis were initially included in the multivariate analysis. Variables included in the final multivariate model for cervical cancer and endometrial cancer were age, race, stage of disease, marital status, chemotherapy, surgery, and radiotherapy.

The site-specific SIRs by radiotherapy status of index cervical and endometrial cancer survivors are shown in Table 3. Regardless of whether patients received RT, the SIRs of HPV-related SPMs in the survivors were higher than those in the general population. Numerically, HPV-related SPM risk was higher in the RT group. The SIR of vaginal cancer was the highest (23.8, 95% CI: 14.9–36.0) in HPV-related SPMs. There were differences in vaginal cancer risk between the SIR of cervical cancer survivors (23.8, 95% CI: 14.9–36.0) and the SIR of endometrial cancer survivors (7.6, 95% CI: 4.8–11.5). The SIR of vulvar cancer was the second highest, and RT increased its incidence in cervical cancer survivors (SIR = 8.3, 95% CI: 5.3–12.4 for RT vs. 3.0, 95% CI: 1.5–5.2 for no RT). The SIR of SPM in oropharyngeal cancers numerically increased in cervical cancer survivors (1.5, 95% CI: 0.6–3.4) but numerically decreased in endometrial cancer survivors (0.72, 95% CI: 0.31–1.35). In addition, the SPM risk in the rectum increased in both cervical and endometrial cancer survivors who received RT (SIR = 2.0, 95% CI: 1.2–3.1 vs. 1.5, 95% CI: 1.1–2.0) but not in patients who did not receive RT (SIR = 0.94, 95% CI: 0.48–1.64 vs. 1.1, 95% CI: 0.9–1.4).

**Table 3:** Potential site SIRs by radiotherapy status of index cervical cancer and endometrial cancer survivors, SEER, 1988–2015.

**Site of second primary malignancy**	**Cervical cancer, RT**		**Endometrial cancer, RT**		**Cervical cancer, no RT**		**Endometrial cancer, no RT**	
	**O/E**	**SIR (95% Cl)^†^**	**O/E**	**SIR (95% Cl)^†^**	**O/E**	**SIR (95% Cl)^†^**	**O/E**	**SIR (95% Cl) ^†^**
HPV-related SPMs	74/20	3.7^*^ (2.9–4.6)	100/62	1.6^*^ (1.3–1.9)	77/28	2.7^*^ (2.2–3.4)	199/176	1.1 (1.0–1.3)
Oropharynx	5/3	1.5 (0.6–3.4)	7/10	0.72 (0.31–1.35)	6/5	1.3 (0.6–2.5)	22/28	0.78 (0.51–1.14)
Female Genital System	49/5	9.7^*^ (7.2–12.8)	48/18	2.7^*^ (2.0–3.6)	50/7	7.1^*^ (5.3–9.4)	81/49	1.6^*^ (1.3–2.0)
Vagina	22/1	23.8^*^ (14.9–36.0)	22/3	7.6^*^ (4.8–11.5)	35/1	29.3^*^ (20.4–40.8)	48/8	6.0^*^ (4.4–8.0)
Vulva	24/3	8.3^*^ (5.3–12.4)	25/11	2.4^*^ (1.5–3.5)	12/4	3.0^*^ (1.5–5.2)	29/29	0.99 (0.66–1.43)
Anus, Anal Canal and Anorectum	½	0.45 (0.01–2.50)	3/7	0.44 (0.09–1.28)	9/4	2.6^*^ (1.2–4.9)	9/20	0.45^*^ (0.21–0.86)
Rectum	19/10	2.0^*^ (1.2–3.1)	42/28	1.5^*^ (1.1–2.0)	12/13	0.94 (0.48–1.64)	86/78	1.1 (0.9–1.4)
Smoking-related SPMs	262/83	3.2^*^ (2.8–3.6)	327/309	1.1 (1.0–1.2)	158/97	1.6^*^ (1.4–1.9)	601/842	0.71^*^ (0.66–0.77)
Esophagus	7/3	2.6^*^ (1.0–5.3)	9/9	0.96 (0.44–1.82)	3/3	0.96 (0.20–2.82)	19/25	0.75 (0.45–1.17)
Lung and Bronchus	213/68	3.1^*^ (2.7–3.6)	244/252	0.97 (0.85–1.10)	134/80	1.7^*^ (1.4–2.0)	464/688	0.67^*^ (0.61–0.74)
Bladder	42/12	3.6^*^ (2.6–4.8)	74/48	1.6^*^ (1.2–2.0)	21/14	1.5 (1.0–2.4)	118/128	0.92 (0.76–1.10)
Hormone-related SPMs	135/178	0.76^*^ (0.63–0.90)	483/500	0.97 (0.88–1.06)	204/272	0.75^*^ (0.65–0.86)	1412/1444	0.98 (0.93–1.03)
Female Breast	110/163	0.68^*^ (0.56–0.82)	472/451	1.1 (1.0–1.1)	190/249	0.76^*^ (0.66–0.88)	1349/1308	1.0 (1.0–1.1)
Ovary	25/16	1.6^*^ (1.1–2.3)	11/48	0.23^*^ (0.12–0.41)	14/23	0.61 (0.33–1.02)	63/136	0.46^*^ (0.36–0.59)

Abbreviations: cSIR: standardized incidence ratio; SPMs: second primary malignancies; RT: received radiotherapy; no RT: not received radiotherapy; CI: confidence interval; O: observed number of cancer patients; E: expected number of cancer patients; HPV: human papillomavirus. ^*^P value of SIR < 0.05 was considered significant. ^†^The SIR and 95% CI was automatically generated by the SEER MP-SIR.

For potentially smoking-related SPMs, the SIR in cervical cancer survivors increased (3.2, 95% CI: 2.8–3.6) but not in endometrial cancer survivors (1.1, 95% CI: 1.0–1.2). The SPM risk of the no-RT group (SIR = 0.71, 95% CI: 0.66–0.77) was even lower than that of the general population. The SIR of the RT group (3.2, 95% CI: 2.8–3.6 and 1.1, 95% CI: 1.0–1.2) was significantly higher than that of the no-RT group (1.6, 95% CI: 1.4–1.9 and 0.71, 95% CI: 0.66–0.77) for both cervical and endometrial cancer survivors. Bladder cancer incidence was the highest in potentially smoking-related SPMs (SIR = 3.6, 95% CI: 2.6–4.8). For potentially hormone-related SPMs, the SIR (0.76, 95% CI: 0.63–0.90) for cervical cancer survivors was lower than that for endometrial cancer survivors. RT did not affect the risk of potentially hormone-related SPMs in either cervical or endometrial cancer survivors.

The multiplicative and additive interactions between HPV, smoking, hormones and RT in potential SPM risk are shown in Table 4. HPV and RT had no interaction effect on potential SPM risk in either cervical or endometrial cancer survivors. However, there were possible significant multiplicative (*P* < 0.001) and additive (*P* < 0.01) interactions between RT and smoking for cervical cancer survivors. The corrected *P* value <0.001 were still considered significant while taking the multiplicity into account (*P* < 0.017). We found that potentially HPV- and smoking-related SPMs each may be individually accounted for only 10.0% and 27.7% of SPMs from cervical cancer, respectively. The possible attribution proportion due to the interaction of RT and smoking for potential SPM risk reached 36%. There was also a possible negative multiplicative interaction (*P* = 0.01) between the proxy of hormones and RT for cervical cancer survivors. Although the possible multiplicative interaction between hormones and RT depends on latency, there was no multiplicative interaction at 60 months in cervical cancer survivors (Supplementary Table 1). In addition, there was no additive interaction, regardless of the latency year. No interaction was observed between any potential risk factors for SPM risk in endometrial cancer survivors.

**Table 4:** Interactions between HPV, smoking, and hormone with RT in the potential risk of SPMs from cervical cancer and endometrial cancer survivors with 12 months latency since the initial primary diagnosis, SEER, 1988–2015.

**Types of interactions**	**Variables ^†^**	**HPV Value (95% Cl), *P***	**Smoking Value (95% Cl), *P***	**Hormone Value (95% Cl), *P***
Multiplicative interactions	RT in cervical cancer	0.97 (0.68–1.39), ^*^P = 0.88	1.7 (1.3–2.3), ^*^P < 0.001	0.70 (0.53–0.92), ^*^P = 0.01
	RT in Endometrial cancer	0.80 (0.62–1.03), ^*^P = 0.09	1.1 (0.9–1.3), ^*^P = 0.5	0.95 (0.81–1.11), ^*^P = 0.52
Additive interactions	RT in cervical cancer	RERI: 0.02 (–0.69–0.73), ^‡^*P* = 0.96 AP: 0.01 (–0.32–0.34), ^‡^*P* = 0.96 S: 1.0 (0.5–1.9), ^‡^*P* =1.0	RERI: 0.87 (0.42–1.31), ^‡^*P* < 0.001 AP: 0.36 (0.21–0.52), ^‡^*P* < 0.001 S: 2.6 (1.3–5.3), ^‡^*P* = 0.006	RERI: –0.59 (–1.22–0.04), ^‡^*P* = 0.07 AP: –0.25 (–0.54–0.05), ^‡^*P* = 0.10 S: 0.70 (0.48–1.03), ^‡^*P* = 0.07
	RT in Endometrial cancer	RERI: –0.46 (–0.90–0.02), ^‡^*P* = 0.04 AP: –0.29 (–0.61–0.03), ^‡^*P* = 0.08 S: 0.57 (0.31–1.03), ^‡^*P* = 0.06	RERI: –0.03 (–0.32–0.27), ^‡^*P* = 0.87 AP: –0.01 (–0.16–0.13), ^‡^*P* = 0.87 S: 0.98 (0.74–1.29), ^‡^*P* = 0.87	RERI: 0.01 (–0.37–0.39), ^‡^*P* = 0.97 AP: 0.00 (–0.11–0.11), ^‡^*P* = 0.97 S: 1.0 (0.9–1.2), ^‡^*P* = 1.0

Abbreviations: SPMs: second primary malignancies; HPV: human papillomavirus; RT: received radiotherapy; no RT: not received radiotherapy; RERI: the relative excess risk due to interaction; AP: the attribution proportion due to interaction; S: the synergy index. ^†^Interaction terms included RT with HPV/smoking/hormone, respectively. ^*^P for interaction was derived from Cox regression analyses. ^‡^*P* for interaction was derived from Cox regression analyses and Anderson’s excel table.

**Figure 2:**
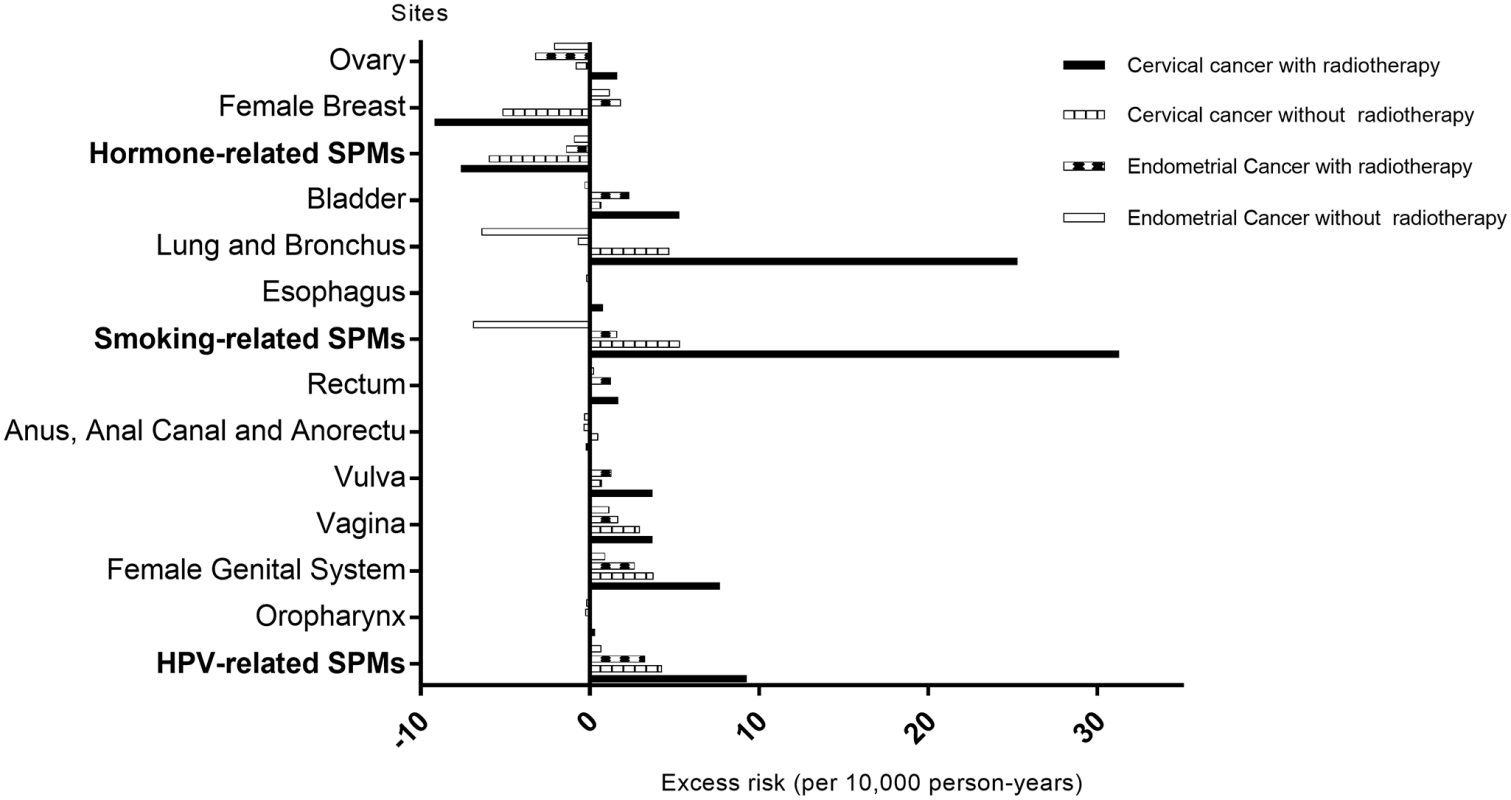
**Excess risk of second primary malignancies (SPMs) by radiotherapy status of index cervical cancer and endometrial cancer survivors.** Abbreviations: SPMs: second primary malignancies; HPV: human papillomavirus.

Figure 2 demonstrates the excess risk of SPMs in cervical and endometrial cancer survivors. Overall, the trend of excess risk was consistent with the corresponding SIRs. The largest difference in SPM excess risk between cervical and endometrial cancer survivors was observed in potentially smoking-related SPMs (31.3 vs. 1.6 per 10,000 person-years). However, the trend was opposite for some sites such as the breast (excess risk: –9.2 vs. 1.8 per 10,000 person-years). Cervical cancer survivors with RT had a substantially higher potential HPV-related SPM excess risk than endometrial cancer survivors (9.3 vs. 3.3 per 10,000 person-years). Although the excess risk of potentially hormone-related SPMs in endometrial cancer survivors was higher than that of cervical cancer survivors (–1.4 vs. –7.6 per 10,000 person-years), it was lower than the general population risk.

## DISCUSSION

To the best of our knowledge, this is the first study to quantify the relative contribution and interactions of possible risk factors for SPMs in cervical cancer survivors compared with endometrial cancer survivors, taking advantage of their anatomical and treatment similarity, and etiological heterogeneity. Potentially HPV and smoking-related SPMs may be accounted for 10.0% and 27.7%, respectively, of SPMs for cervical cancer survivors. Moreover, the most possible significant difference in the risk of SPMs was in smoking-related sites. Strong synergistic interactions between RT and smoking were also observed (*P*_interaction_ < 0.001), accounting for 36% of potentially smoking-related SPMs in cervical cancer survivors in our analyses.

There was an increased risk of potentially smoking-related SPMs in cervical cancer survivors, similar to the conclusion of Underwood et al., who found that the risk of smoking-related SPMs in cervical cancer survivors was almost twice that of breast and colorectal cancer survivors [9]. The reason may be that the estimated smoking prevalence of cervical cancer patients is over 40%, while it is only 14% and 12% in breast and colorectal cancer survivors [9], respectively. In our study, the difference in SIR and the excess risk in smoking-related sites was most significant among the potentially HPV-, smoking-, and hormone-related SPM subgroups. The differences in SPM risk could be predominantly correlated to the high smoking rate of cervical cancer survivors. This finding highlights the importance of smoking cessation in the prevention of SPMs for cervical cancer. However, the SPM risk in smoking-related sites for endometrial cancer survivors decreased (SIR: 0.71, 95% CI: 0.66–0.77), consistent with results from Zhou et al. [25]. Smoking might decrease the SPM risk of endometrial cancer survivors by reducing body fat and exerting potent antiestrogenic effects [6].

Etiologically, cervical cancer is also associated with HPV infection, unlike endometrial cancer. HPV may affect susceptibility to SPMs for cervical cancer survivors [9]. This effect could, at least in part, explain why a higher SIR was observed in cervical cancer survivors. The overall SIR of cervical cancer survivors in potentially HPV-related sites was significantly higher than that of endometrial cancer survivors (SIR: 3.7 vs. 1.6) regardless of RT. For example, the SIR of SPMs in cervical cancer patients numerically increased, while that in endometrial cancer patients numerically decreased in oropharyngeal cancers. Although smoking and alcohol consumption are the main risk factors for oropharyngeal cancers, as smoking rates decline in the United States, the increased incidence of oropharyngeal cancers has been attributed to high-risk HPV infection [9, 26]. Therefore, the differences in SIR between the two cancers might be attributed to the prevalence of HPV and/or interactions between the above risk factors [23, 26]. In addition, hormone-related factors such as obesity, nulliparity, late menopause, use of tamoxifen, and diabetes mellitus affect endometrial but not cervical cancer [12, 13]. This difference, at least in part, accounted for the higher SIR in potentially hormone-related SPMs of endometrial versus cervical cancer survivors. Thus, in addition to smoking, the differences between SPM risk of cervical and endometrial cancer survivors might also be related to HPV infection and hormonal status.

Radiotherapy is essential for the local control of both cervical and endometrial cancers. However, this treatment can also result in SPMs [6]. It was estimated that approximately 8% of SPMs in cancer survivors were due to radiation therapy [14]. The highest incidence rate was between 4 and 15 years after RT [16, 27]. Patients who received RT had a higher SPM risk in this study, with the highest risk found in the vagina of both cervical and endometrial cancer survivors, similar to previous reports [6, 28]. In SEER database, second vagina cancer was defined as a primary cancer, unless a pathologist compares the second tumor to the primary tumor and states that second tumor is a recurrence of cancer from the previous primary. In addition, Stage IV patients were excluded in this study to avoid metastases. Moreover, SPMs predominantly occur in irradiated or adjacent areas because of the local effect of RT [17, 28]. RT thus plays an important role in the highest SPM risk of second vaginal cancer. This result suggests that survivors, especially smokers or those exposed to second-hand smoke, who have received RT are encouraged to be monitored more frequently to facilitate early detection of SPMs.

Notably, radiotherapy of the pelvis can result in ovarian insufficiency since ovarian tissue is sensitive to radiation [29]. There was decreased breast SPM risk (SIR = 0.68, 95% CI: 0.56–0.82) in cervical cancer survivors, possibly due to alterations in hormone levels in the breast tissue following hysterectomy, ovariectomy, and premature ovarian failure resulting from radiation [19, 30]. Sensitivity analyses were conducted for survivors who received hysterectomy and/or ovariectomy, and no significant difference was observed. Thus, the results of hormone-related SPMs are robust.

However, none of these factors alone can fully explain the differences in potential SPM risk between cervical and endometrial cancer survivors. Recent studies have shown that risk factors associated with SPMs can jointly interact [19, 31]. There were potentially strong synergistic interactions between RT and smoking (*P* < 0.01) in cervical cancer survivors in this study. Despite some confounders existed, the significant interaction could effectively rule out most of the confounding factors. For causative exposures, the positive multiplicative positive additive was the strongest form of interaction [22]. In this case, each exposure amplifies the causative effects of the other on either the additive or the multiplicative scale [22]. Although the findings were similar to those of Lois B et al., who concluded that the SPM risk from smoking and treatment was compatible with a multiplicative relation [32], a positive additive interaction was further identified in this study, indicating a potential biological interaction [33]. Moreover, the interaction was biologically feasible because both tobacco carcinogens and radiation can result in genetic mutations that may jointly contribute to SPM formation [34, 35]. There was also a possible negative multiplicative interaction (*P* = 0.01) between hormones and RT. This finding can also be mechanistically explained by the ovarian insufficiency caused by radiotherapy to the pelvis [29]. There were no interactions between HPV and RT in SPM risk. The interaction between smoking and HPV in SPM risk could not be determined due to insufficient available data. However, it has been reported that smoking increases HPV viral load and is associated with the persistence of high-risk HPV [9, 35]. Mechanisms have been suggested that smoking increases HPV replication and DNA damage in epithelial cells and affects both innate and adaptative immune responses against HPV [36]. Smoking contributes to the development of HPV-related cancer [37].

Overall, smoking was likely the most prominent factor in the SPM risk for cervical cancer survivors, followed by RT and HPV [14, 31]. Notably, many cancer survivors still consume tobacco after diagnosis and treatment [15, 38, 39], exposing themselves to SPM risk. Thus, smoking cessation is an effective method to prevent SPMs in cancer survivors [31, 39]. In addition, HPV monitoring and management are recommended for cervical cancer survivors. Moreover, protecting the surrounding normal tissue by using advanced radiotherapy techniques and regular SPM screening after radiotherapy is critical. Early detection can improve the lifespan of survivors, and SPMs are preventable if lesions are detected early and adequately managed [11]. Therefore, although further research is necessary, our findings are of important implications for second primary malignancies surveillance and prevention. Specifically, our results advocated that 1) smoking cessation should be implemented among cervical cancer survivors, especially for those who received RT. 2) increased surveillance could be done. In practice, survivors may be followed-up every three months during the first two years after successful treatment for cervical cancer and then twice yearly and thereafter for the rest of their lives [4]. For those regions exposed to radiotherapy- or smoking-related sites, physical or other examinations (for example, vaginal examination) should be more frequent than previously assumed. We think it is prudent to propose twice-yearly followed-up for the rest of their lives and at least a chest CT, an abdominal and pelvic MRI should be recommended for every surveillance. However, additional researches on the frequency and detailed surveillance strategies should be carried out. For endometrial cancer survivors, although they still should stick to once-a-year routine checkup, but breast examination could be on the checklist. 3) improvement of the HPV vaccine reduces the incidence of cervical cancer, which will decrease the number of cervical cancer survivors naturally and thus also the HPV-related SPMs. Hence, it is important to promote HPV vaccine uptake for the primary prevention of HPV-related cancers and also HPV-related SPMs [40].

This study has some undeniable limitations. First, the SEER registry provided no information on HPV infection, details of smoking habits, RT doses, or changes in treatments over time. We used the grouping of HPV-, smoking- or hormone-related cancer patients as proxies for SPM risk factors. There is no information in most large databases about risk factors such as HPV infection, smoking or exposure to hormones, etc., and there is a certain difficulty to collect all the above information in the real world. However, we used innovative study designs to circumvent this pitfall. Approaches that rely on proxies have a potential advantage when data are difficult to collect but require evidence supporting their accuracy. The proxy for HPV might be accurate enough because approximately 80% of HPV-related cancers are caused by HPV [18, 38]; the proxy for smoking- or hormone-related cancers might be inaccurate because the proportion of malignancies caused by these risk factors are highly uncertain and there may be significant unmeasured confounders. Therefore, we tested the hypotheses by interaction analyses, which effectively ruled out most of the confounding. The significant interaction that we found strongly indicates a true role of these risk factors in SPM risk. In addition, we did conduct sensitivity analyses by classifying mouth, throat, larynx, esophagus, lung, liver, stomach, pancreas, bladder, kidney, prostate, colon, and rectum as potentially smoking-related SPMs, and we found the positive multiplicative positive additive interactions between RT and smoking (*P* = 0.004, Supplementary Table 2) still exist, even after Bonferroni correction. Second, we head-to-head compared and comprehensively evaluated the different associations of the SPMs risk factors between survivors of cervical cancer and endometrial cancer, taking the advantage of their anatomical and treatment similarity and etiological heterogeneity. Although cervical cancer and endometrial cancer are tumors with completely different pathologic mechanisms and oncological properties, they have a close anatomic location and similar treatment especially radiotherapy. This comparison provides a unique opportunity to delineate the relative contribution and interactions of treatment, cancer, and patient-related determinants to second primary malignancies risks. Third, chemotherapy agents and anti-estrogen therapy may also affect the SPM outcomes. Chemotherapy regimens for cervical and endometrial cancer are usually cisplatin-based [41]. However, a meta-analysis showed that cisplatin was not associated with an increased risk for second cancers. It is likely that cisplatin is not a remarkable confounding factor affecting the SPM outcomes. Anti-estrogen therapy is mainly for endometrial cancer not cervical cancer. In addition, there was no interaction between RT and hormone proxy, therefore, its impact on the risk of SPMs of cervical cancer survivors may not be remarkable. In addition, sensitivity analyses between the two cancer survivors (Supplementary Tables 1–11) were conducted to assess the robustness of the results.

In conclusion, large population-database analyses suggested that RT, HPV, and smoking promoted SPMs in cervical cancer survivors at different magnitudes. There were possible strong synergistic interactions between smoking and RT. RT also increased the SPM risk in endometrial cancer survivors. Although future studies are warranted, it is prudent to suggest smoking cessation, HPV monitoring and management, and increased surveillance of cancer survivors as critical considerations for SPM prevention, especially in those who receive RT.

## Supplementary Materials

Supplementary Tables
