# Supplementary material for: Second primary malignancies in cervical cancer and endometrial cancer survivors: a population-based analysis
Source: Aging (Albany NY). 2022 May 4;14(9):3836–55. doi: 10.18632/aging.204047 (PMC9134942; doi:10.18632/aging.204047)
Supplement: Supplementary Tables [file aging-14-204047-s001.pdf]

SUPPLEMENTARY TABLES

Supplementary Table 1. Interactions between HPV, smoking, and hormone with RT in the potential risk of SPMs from cervical cancer and endometrial cancer survivors with 60 months latency since the initial primary diagnosis, SEER, 1988–2015.

Types of interactions	Variables[$]	HPV<br>Value (95% Cl), *P*	Smoking<br>Value (95% Cl), *P*	Hormone<br>Value (95% Cl), *P*
Multiplicative interactions	RT in cervical cancer	1.1 (0.7–1.7), $^{*}P = 0.7$	1.5 (1.1–2.1), $^{*}P = 0.017$	0.76 (0.55–1.07), $^{*}P = 0.12$
	RT in Endometrial cancer	0.74 (0.53–1.03), $^{*}P = 0.07$	1.1 (0.8–1.3), $^{*}P = 0.6$	0.90 (0.73–1.11), $^{*}P = 0.33$
Additive interactions	RT in cervical cancer	RERI: 0.20 (–0.70–1.09), $^{\#}P = 0.67$<br>AP: 0.09 (–0.29–0.46), $^{\#}P = 0.65$<br>S: 1.2 (0.6–2.5), $^{\#}P = 0.7$	RERI: 0.55 (0.07–1.03), $^{\#}P = 0.03$<br>AP: 0.27 (0.06–0.49), $^{\#}P = 0.01$<br>S: 2.2 (0.9–5.5), $^{\#}P = 0.1$	RERI: –0.49 (–1.28–0.31), $^{\#}P = 0.23$<br>AP: –0.19 (–0.52–0.15), $^{\#}P = 0.27$<br>S: 0.77 (0.49–1.19), $^{\#}P = 0.24$
	RT in Endometrial cancer	RERI: –0.62 (–1.16–0.07), $^{\#}P = 0.03$<br>AP: –0.44 (–0.90–0.03), $^{\#}P = 0.06$<br>S: 0.40 (0.15–1.04), $^{\#}P = 0.06$	RERI: –0.11 (–0.50–0.29), $^{\#}P = 0.60$<br>AP: –0.05 (–0.25–0.15), $^{\#}P = 0.61$<br>S: 0.90 (0.62–1.32), $^{\#}P = 0.60$	RERI: –0.21 (–0.72–0.31), $^{\#}P = 0.43$<br>AP: –0.06 (–0.21–0.09), $^{\#}P = 0.44$<br>S: 0.92 (0.76–1.13), $^{\#}P = 0.43$

Abbreviations: HPV: human papillomavirus; RT: radiotherapy; no RT: not received radiotherapy; SPMs: second primary malignancies; RERI: the relative excess risk due to interaction; AP: the attribution proportion due to interaction; S: the synergy index. [$]Interaction terms included RT with HPV/smoking/hormone, respectively. $^{*}P$ for interaction was derived from Cox regression analyses. $^{\#}P$ for interaction was derived from Cox regression analyses and Anderson's excel table.

Supplementary Table 2. Interactions between smoking with RT in the potential risk of SPMs from cervical cancer and endometrial cancer survivors with 12 months latency since the initial primary diagnosis, SEER, 1988–2015.

Types of interactions	Variables	Smoking[1]<br>Value (95% Cl), *P*	Smoking[2]<br>Value (95% Cl), *P*
Multiplicative interactions	RT in cervical cancer	1.7 (1.3–2.3), $^{*}P < 0.001$	1.5 (1.1–1.9), $^{*}P = 0.004$
	RT in endometrial cancer	1.1 (0.9–1.3), $^{*}P = 0.5$	1.0 (0.9–1.2), $^{*}P = 0.7$
Additive interactions	RT in cervical cancer	RERI: 0.87 (0.42–1.31), $^{‡}P < 0.001$<br>AP: 0.36 (0.21–0.52), $^{‡}P < 0.001$<br>S: 2.6 (1.3–5.3), $^{‡}P = 0.006$	RERI: 0.54 (0.24–0.84), $^{‡}P < 0.001$<br>AP: 0.32 (0.15–0.49), $^{‡}P < 0.001$<br>S: 5.1 (0.5–55.1), $^{‡}P = 0.18$
	RT in endometrial cancer	RERI: –0.03 (–0.32–0.27), $^{‡}P = 0.87$<br>AP: –0.01 (–0.16–0.13), $^{‡}P = 0.87$<br>S: 0.98 (0.74–1.29), $^{‡}P = 0.87$	RERI: 0.03 (–0.11–0.17), $^{‡}P = 0.68$<br>AP: 0.03 (–0.13–0.20), $^{‡}P = 0.68$<br>S: 0.81 (0.32–2.09), $^{‡}P = 0.67$

Abbreviations: SPMs: second primary malignancies; SEER: surveillance, epidemiology, and end results; RT: received radiotherapy; RERI: the relative excess risk due to interaction; AP: the attribution proportion due to interaction; S: the synergy index. $^{*}P$ for interaction was derived from Cox regression analyses. $^{‡}P$ for interaction was derived from Cox regression analyses and Anderson's excel table. [1]Cancers of the esophagus, lung, bronchus, and bladder were classified as potentially smoking-related SPMs. [2]Cancers of the mouth, throat, larynx, esophagus, lung, liver, stomach, pancreas, bladder, kidney, prostate, colon, and rectum were classified as potentially smoking-related SPMs.

Supplementary Table 3. Potential site SIRs by radiotherapy status of index cervical cancer and endometrial cancer survivors, SEER, 1988–2015, with 60 months latency since the initial primary diagnosis.

Site of second primary malignancy	Cervical cancer, RT		Endometrial cancer, RT		Cervical cancer, no RT		Endometrial cancer, no RT	
	O/E	SIR (95% Cl)[#]	O/E	SIR (95% Cl)[#]	O/E	SIR (95% Cl)[#]	O/E	SIR (95% Cl)[#]
HPV-related SPMs	46/13	3.5* (2.3–5.0)	61/35	1.8* (1.2–2.3)	57/22	2.6* (1.9–3.3)	112/110	1.0 (0.8–1.3)
Oropharynx	3/2	1.4 (0.3–4.8)	6/6	1.0 (0.4–2.4)	3/4	0.79 (0.28–2.13)	13/18	0.74 (0.40–1.46)
Female Genital System	30/3	8.9* (6.0–12.7)	29/11	2.6* (1.8–3.8)	37/6	6.6* (4.6–9.1)	33/32	1.0 (0.7–1.4)
Vagina	10/1	16.6* (7.9–30.4)	13/2	7.4* (3.9–12.6)	25/1	26.7* (17.3–39.5)	14/5	2.7* (1.5–4.6)
Vulva	17/2	8.9* (5.2–14.2)	15/7	2.3* (1.3–3.7)	18/19	3.1* (1.5–5.8)	18/19	0.94 (0.56–1.48)
Anus, Anal Canal and Anorectum	0/2	0.00 (0.00–2.43)	1/4	0.24 (0.01–1.35)	7/3	2.4 (1.0–5.0)	5/13	0.40* (0.13–0.92)
Rectum	13/6	2.1* (1.1–3.6)	25/16	1.6* (1.0–2.3)	10/10	0.99 (0.48–1.82)	60/48	1.3 (1.0–1.6)
Smoking-related SPMs	139/55	2.5* (2.1–3.0)	188/188	1.0 (0.9–1.2)	119/77	1.5* (1.3–1.8)	340/546	0.62* (0.56–0.69)
Esophagus	3/2	1.7 (0.4–4.9)	6/6	1.1 (0.4–2.3)	3/2	1.2 (0.3–3.6)	9/16	0.55 (0.25–1.05)
Lung and Bronchus	115/46	2.5* (2.1–3.0)	130/152	0.85 (0.71–1.01)	100/64	1.6* (1.3–1.9)	259/445	0.58* (0.51–0.66)
Bladder	21/8	2.7* (1.7–4.1)	52/30	1.8* (1.3–2.3)	16/11	1.5 (0.9–2.4)	72/84	0.85 (0.67–1.08)
Hormone-related SPMs	94/115	0.82* (0.66–1.00)	276/286	0.96 (0.85–1.08)	157/212	0.74* (0.63–0.87)	834/880	0.95 (0.88–1.01)
Female Breast	77/105	0.73* (0.58–0.92)	272/259	1.1 (0.9–1.2)	147/194	0.76* (0.64–0.89)	808/797	1.0 (1.0–1.1)
Ovary	17/10	1.7 (1.0–2.7)	4/28	0.14* (0.04–0.37)	10/18	0.56 (0.27–1.03)	26/83	0.31* (0.20–0.46)

Abbreviations: SIR: standardized incidence ratio; SEER: surveillance, epidemiology, and end results; SPMs: second primary malignancies; RT: radiotherapy; no RT: not received radiotherapy; CI: confidence interval; O: observed number of cancer patients; E: expected number of cancer patients; HPV: human papillomavirus. *P value of SIR < 0.05 was considered significant. [#]The 95% CI was automatically generated by the SEER MP-SIR.

Supplementary Table 4. Potential site SIRs by radiotherapy status of index cervical cancer and endometrial cancer survivors received hysterectomy and/or ovariectomy, SEER, 1988–2015, with 12 months latency since the initial primary diagnosis.

Site of second primary malignancy	Cervical cancer, RT		Endometrial cancer, RT		Cervical cancer, no RT		Endometrial cancer, no RT	
	O/E	SIR (95% CI)#	O/E	SIR (95% CI)#	O/E	SIR (95% CI)#	O/E	SIR (95% CI)#
HPV-related SPMs	24/6	3.7* (2.3–5.4)	99/60	1.6* (1.3–1.9)	55/22	2.5* (1.9–3.2)	191/172	1.1 (1.0–1.3)
Oropharynx	1/1	0.85 (0.12–4.36)	7/10	0.70 (0.30–1.38)	4/3	1.2 (0.5–2.7)	19/30	0.63 (0.39–1.02)
Female Genital System	16/2	9.8* (5.6–16.0)	47/17	2.7* (2.0–3.6)	37/6	6.8* (4.8–9.3)	80/48	1.7* (1.3–2.1)
Vagina	7/1	24.6* (9.9–50.6)	21/3	7.5* (4.6–11.4)	29/1	31.6* (21.2–45.4)	47/8	6.1* (4.5–8.1)
Vulva	9/1	9.8* (4.5–18.6)	25/10	2.4* (1.6–3.6)	5/3	1.6 (0.5–3.8)	29/28	1.0 (0.7–1.5)
Anus, Anal Canal and Anorectum	0/1	0.00 (0.00–4.82)	3/7	0.45 (0.09–1.31)	4/3	1.5 (0.4–3.7)	9/19	0.47* (0.21–0.69)
Rectum	8/3	2.7* (1.2–5.3)	41/27	1.5* (1.1–2.1)	10/10	1.0 (0.5–1.9)	83/75	1.1 (0.7–1.4)
Smoking-related SPMs	64/25	2.6* (2.0–3.3)	315/301	1.1 (1.0–1.2)	126/76	1.7* (1.4–2.0)	582/810	0.72* (0.66–0.78)
Esophagus	1/1	1.3 (0.0–7.0)	9/9	0.99 (0.45–1.88)	1/2	0.42 (0.01–2.32)	18/24	0.74 (0.44–1.17)
Lung and Bronchus	0/1	0.00 (0.00–5.94)	11/9	1.3 (0.6–2.3)	108/63	1.7* (1.4–2.1)	12/23	0.53* (0.27–0.92)
Bladder	14/4	4.0* (2.2–6.8)	71/46	1.5* (1.2–1.9)	17/11	1.6 (0.9–2.6)	115/124	0.93 (0.77–1.12)
Hormone-related SPMs	41/60	0.68* (0.49–0.92)	472/486	0.97 (0.89–1.06)	155/212	0.73* (0.62–0.85)	1360/1389	0.98 (0.93–1.03)
Female Breast	36/55	0.66* (0.46–0.91)	462/439	1.1 (1.0–1.2)	145/195	0.75* (0.63–0.88)	1308/1258	1.0 (0.9–1.1)
Ovary	5/5	0.96 (0.31–2.24)	10/46	0.22* (0.10–0.40)	10/18	0.55 (0.27–1.02)	52/131	0.40* (0.30–0.52)

Abbreviations: SIR: standardized incidence ratio; SEER: surveillance, epidemiology, and end results; RT: radiotherapy; no RT: not received radiotherapy; CI: confidence interval; SPMs: second primary malignancies; O: observed number of cancer patients; E: expected number of cancer patients; HPV: human papillomavirus. $Hysterectomy and/or ovariectomy: total hysterectomy, modified radical or extended hysterectomy, radical hysterectomy, extended radical hysterectomy with/without removal of tubes and ovaries. *P value of SIR < 0.05 was considered significant. #The 95% CI was automatically generated by the SEER MP-SIR.

Supplementary Table 5. Potential site SIRs sub-grouped by median age of index cervical cancer and endometrial cancer survivors, SEER, 1988–2015, with 12 months latency since the initial primary diagnosis.

Site of second primary malignancy	Cervical cancer, age$ < 44		Cervical cancer, age$ ≥ 44		Endometrial cancer, age& < 62		Endometrial cancer, age& ≥ 62	
	O/E	SIR (95% CI)#	O/E	SIR (95% CI)#	O/E	SIR (95% CI)#	O/E	SIR (95% CI)#
HPV-related SPMs	45/15	3.0* (2.0–4.3)	106/33	3.2* (2.5–4.1)	125/97	1.3* (1.0–1.6)	174/141	1.2* (1.1–1.4)
Oropharynx	3/3	1.2 (0.2–3.8)	8/5	1.5 (0.6–3.2)	16/18	0.90 (0.44–1.58)	13/20	0.64 (0.32–1.18)
Female Genital System	32/4	8.7* (6.0–12.3)	67/8	7.9* (6.1–10.1)	49/25	2.0* (1.5–2.6)	81/42	1.9* (1.5–2.4)
Vagina	18/1	32.0* (19.0–50.6)	39/2	25.0* (17.8–34.1)	28/4	7.2* (4.8–10.4)	42/7	5.9* (4.3–8.0)
Vulva	13/2	6.1* (3.2–10.4)	23/5	4.8* (3.0–7.2)	18/13	1.4 (0.8–2.1)	37/27	1.4 (1.0–1.9)
Anus, Anal Canal and Anorectum	4/2	1.9 (0.5–4.7)	6/4	1.7 (0.6–3.6)	6/13	0.46* (0.17–1.00)	6/14	0.43* (0.16–0.94)
Rectum	7/7	1.1 (0.4–2.2)	26/16	1.6* (1.0–2.4)	54/41	1.3 (1.0–1.7)	77/66	1.2 (0.9–1.5)
Smoking-related SPMs	85/30	2.9* (2.3–3.6)	336/151	2.2* (2.0–2.5)	303/390	0.78* (0.69–0.87)	631/767	0.82* (0.76–0.89)
Esophagus	1/1	0.99 (0.02–5.50)	9/5	1.9 (0.9–3.5)	5/11	0.46 (0.15–1.07)	24/24	1.0 (0.6–1.5)
Lung and Bronchus	66/25	2.7* (2.1–3.4)	281/125	2.3* (2.0–2.5)	236/327	0.72* (0.63–0.82)	474/619	0.77* (0.70–0.84)
Bladder	18/4	4.4* (2.6–7.0)	46/21	2.2* (1.6–2.9)	62/53	1.2 (0.9–1.5)	133/125	1.1 (0.9–1.3)
Hormone-related SPMs	136/164	0.83* (0.69–0.98)	204/288	0.71* (0.62–0.81)	825/928	0.89* (0.83–0.95)	1081/1027	1.1 (1.0–1.1)
Female Breast	121/152	0.80* (0.66–0.95)	179/261	0.69* (0.59–0.79)	786/848	0.93* (0.86–0.99)	1046/922	1.1* (1.1–1.2)
Ovary	15/13	1.2 (0.7–1.9)	25/26	0.95 (0.61–1.40)	39/80	0.49* (0.35–0.67)	35/105	0.33* (0.23–0.46)

Abbreviations: SIR: standardized incidence ratio; SEER: surveillance, epidemiology, and end results; CI: confidence interval; O: observed number of cancer patients; E: expected number of cancer patients; SPMs: second primary malignancies; HPV: human papillomavirus. $Median age in cervical cancer was 44 years old. &Median age in endometrial cancer was 62 years old. *P value of SIR < 0.05 was considered significant. #The 95% CI was automatically generated by the SEER MP-SIR.

Supplementary Table 6. Potential site SIRs sub-grouped by different latency of index cervical cancer survivors, SEER, 1988–2015.

Site of second primary malignancy	12–35 months		36–59 months		60–119 months		120+ months	
	O/E	SIR (95% CI)#	O/E	SIR (95% CI)#	O/E	SIR (95% CI)#	O/E	SIR (95% CI)#
HPV-related SPMs	34/6	5.1* (3.5–7.0)	14/5	2.5* (1.4–4.0)	45/12	3.7* (2.9–5.1)	55/22	2.5* (2.0–3.3)
Oropharynx	4/1	3.5* (1.1–8.2)	2/1	1.7 (0.3–6.0)	1/2	0.48 (0.11–2.23)	6/4	1.5 (0.6–3.1)
Female Genital System	25/2	14.9* (9.7–22.1)	7/1	4.8* (2.0–10.0)	35/3	11.2* (7.8–15.5)	32/6	5.5* (3.7–7.7)
Vagina	20/0	63.7* (38.9–98.5)	2/0	7.5 (1.0–27.0)	19/1	33.3* (20.0–52.0)	16/1	16.4* (9.4–26.7)
Vulva	4/1	4.1* (1.1–10.6)	5/1	6.0* (1.9–13.9)	14/2	7.6* (4.2–12.8)	13/3	4.0* (2.1–6.8)
Anus, Anal Canal and Anorectum	2/1	2.8 (0.3–10.2)	1/1	1.6 (0.0–8.9)	2/1	1.4 (0.2–5.0)	5/3	1.7 (0.6–3.9)
Rectum	4/3	1.2 (0.3–3.1)	5/3	1.8 (0.6–4.1)	10/6	1.6 (0.8–3.0)	14/10	1.4 (0.8–2.3)
Smoking-related SPMs	98/26	3.8* (3.1–4.7)	65/22	3.0* (2.3–3.8)	101/48	2.1* (1.7–2.6)	157/85	1.8* (1.6–2.2)
Esophagus	2/1	2.3 (0.3–8.1)	2/1	2.7 (0.3–9.7)	2/2	1.3 (0.2–4.6)	4/3	1.5 (0.4–3.6)
Lung and Bronchus	81/21	3.8* (3.1–4.8)	51/18	2.8* (2.1–3.7)	95/39	2.4* (2.0–3.0)	120/71	1.7* (1.4–2.0)
Bladder	15/4	4.1* (2.3–6.8)	12/3	3.9* (2.0–6.7)	4/7	0.59 (0.16–1.52)	33/12	2.8* (1.9–3.9)
Hormone-related SPMs	48/66	0.72* (0.53–0.96)	40/57	0.70* (0.50–0.95)	117/124	0.95 (0.78–1.14)	135/205	0.66* (0.55–0.78)
Female Breast	41/60	0.68* (0.49–0.92)	35/52	0.67* (0.47–0.93)	102/113	0.90 (0.74–1.10)	122/187	0.65* (0.54–0.78)
Ovary	7/6	1.2 (0.5–2.4)	5/5	0.99 (0.32–2.30)	15/11	1.4 (0.8–2.3)	13/17	0.75 (0.40–1.28)

Abbreviations: SIR: standardized incidence ratio; SEER: surveillance, epidemiology, and end results; CI: confidence interval; O: observed number of cancer patients; E: expected number of cancer patients; SPMs: second primary malignancies; HPV: human papillomavirus. *P value of SIR <0.05 was considered significant. #The 95% CI was automatically generated by the SEER MP-SIR.

Supplementary Table 7. Potential site SIRs sub-grouped by different latency of index endometrial cancer survivors, SEER, 1988–2015.

Site of second primary malignancy	12–35 months		36–59 months		60–119 months		120+ months	
	O/E	SIR (95% CI)#	O/E	SIR (95% CI)#	O/E	SIR (95% CI)#	O/E	SIR (95% CI)#
HPV-related SPMs	72/49	1.4* (1.1–1.8)	49/39	1.2 (0.9–1.6)	92/78	1.2 (1.0–1.5)	83/71	1.2 (0.9–1.4)
Oropharynx	2/8	0.21* (0.0–0.7)	8/7	1.1 (0.6–2.2)	12/13	0.95 (0.54–1.59)	8/11	0.76 (0.38–1.41)
Female Genital System	42/13	3.3* (2.4–4.4)	25/11	2.3* (1.5–3.3)	27/21	1.3 (0.8–1.8)	36/22	1.6* (1.1–2.3)
Vagina	33/2	0.70 (0.47–1.01)	10/2	5.4* (2.6–9.9)	13/4	3.7* (2.0–6.3)	14/3	4.1* (2.3–6.9)
Vulva	9/8	15.2* (10.5–21.3)	12/7	1.8 (1.0–3.2)	12/13	0.94 (0.49–1.65)	22/13	1.7* (1.0–2.5)
Anus, Anal Canal and Anorectum	3/5	0.55 (0.11–1.60)	3/5	0.64 (0.13–1.88)	5/9	0.57 (0.19–1.34)	1/8	0.12* (0.00–0.69)
Rectum	27/23	1.2 (0.8–1.7)	17/19	0.87 (0.51–1.40)	49/35	1.4* (1.0–1.9)	38/29	1.3 (0.9–1.8)
Smoking-related SPMs	207/225	0.92 (0.80–1.05)	195/195	1.0 (0.9–1.2)	312/375	0.83* (0.74–0.93)	220/362	0.61* (0.53–0.69)
Esophagus	7/7	1.0 (0.4–2.1)	6/6	1.0 (0.4–2.2)	10/11	0.88 (0.42–1.62)	6/11	0.56 (0.20–1.21)
Lung and Bronchus	161/185	0.87 (0.74–1.02)	347/322	1.1 (1.0–1.2)	230/306	0.75* (0.66–0.85)	160/294	0.54* (0.46–0.63)
Bladder	39/33	1.2 (0.8–1.6)	30/29	1.0 (0.7–1.5)	72/57	1.3 (1.0–1.6)	54/57	0.94 (0.71–1.23)
Hormone-related SPMs	428/426	1.0 (0.9–1.1)	363/355	1.0 (0.9–1.1)	650/634	1.0 (1.0–1.1)	465/539	0.86* (0.79–0.95)
Female Breast	400/387	1.0 (0.9–1.1)	347/322	1.1 (1.0–1.2)	632/574	1.1* (1.0–1.2)	453/487	0.93 (0.85–1.02)
Ovary	28/40	1.2 (0.5–2.4)	16/33	0.48* (0.27–0.78)	18/60	0.30* (0.18–0.47)	12/51	0.23* (0.12–.0.41)

Abbreviations: SIR: standardized incidence ratio; SEER: surveillance, epidemiology, and end results; CI: confidence interval; O: observed number of cancer patients; E: expected number of cancer patients; SPMs: second primary malignancies; HPV: human papillomavirus. *P value of SIR <0.05 was considered significant. #The 95% CI was automatically generated by the SEER MP-SIR.

Supplementary Table 8. Potential site SIRs sub-grouped by stage of index cervical cancer and endometrial cancer survivors, SEER, 1988–2015, with 12 months latency since the initial primary diagnosis.

Site of second primary malignancy	Cervical cancer, stage I		Cervical cancer, stage II-III		Endometrial cancer, stage I		Endometrial cancer, stage II–III	
	O/E	SIR (95% Cl)#	O/E	SIR (95% Cl)#	O/E	SIR (95% Cl)#	O/E	SIR (95% Cl)#
HPV-related SPMs	80/25	3.0* (2.4–3.8)	38/10	3.5* (2.5–4.7)	201/181	1.1 (1.0–1.3)	62/26	2.3* (1.8–3.0)
Oropharynx	9/4	2.1 (1.1–3.6)	1/1	0.60 (0.20–2.61)	23/32	0.72 (0.47–1.05)	4/4	0.96 (0.33–2.22)
Female Genital System	50/7	7.3* (5.5–9.7)	26/3	9.6* (6.3–14.0)	80/51	1.6* (1.3–2.0)	35/8	4.6* (3.2–6.4)
Vagina	32/1	27.5* (18.8–38.8)	15/0	30.9* (17.3–50.9)	37/8	4.5* (3.2–6.2)	24/1	19.2* (12.3–28.5)
Vulva	15/4	3.9* (2.2–6.4)	10/2	6.5* (3.1–11.9)	39/30	1.3 (0.9–1.8)	10/4	2.2* (1.1–4.1)
Anus, Anal Canal and Anorectum	6/3	1.8 (0.7–3.9)	0/1	0.00 (0.00–3.01)	8/20	0.39* (0.17–0.78)	1/3	0.32 (0.01–1.76)
Rectum	15/12	1.2 (0.7–2.0)	11/5	2.2* (1.1–3.9)	90/80	1.1 (0.9–1.4)	22/12	1.8* (1.1–2.8)
Smoking-related SPMs	178/97	1.8* (1.6–2.1)	140/44	3.2* (2.7–3.8)	637/869	0.73* (0.68–0.79)	161/130	1.2* (1.1–1.5)
Esophagus	3/3	0.96 (0.20–2.8)	4/1	2.8 (0.8–7.3)	21/26	0.81 (0.50–1.23)	4/4	1.0 (0.3–2.6)
Lung and Bronchus	145/80	1.8* (1.5–2.1)	112/36	3.1* (2.6–3.7)	487/710	0.69* (0.63–0.75)	120/106	1.1 (0. 9–1.4)
Bladder	30/14	2.2* (1.5–3.2)	24/6	3.9* (2.5–5.8)	129/133	0.97 (0.81–1.15)	37/20	1.9* (1.3–2.6)
Hormone-related SPMs	194/259	0.75* (0.65–0.86)	74/97	0.76* (0.60–0.96)	1447/1464	0.99 (0.94–1.04)	213/225	0.95 (0.83–1.09)
Female Breast	176/237	0.74* (0.64–0.86)	60/88	0.68* (0.52–0.87)	1402/1326	1.1* (1.0–1.1)	204/204	1.0 (0.9–1.2)
Ovary	18/22	0.81 (0.48–1.29)	14/9	1.6 (0.9–2.8)	45/138	0.33* (0.24–0.44)	9/21	0.43* (0.20–0.82)

Abbreviations: SIR: standardized incidence ratio; SEER: surveillance, epidemiology, and end results; CI: confidence interval; O: observed number of cancer patients; E: expected number of cancer patients; SPMs: second primary malignancies; HPV: human papillomavirus. *P value of SIR <0.05 was considered significant. #The 95% CI was automatically generated by the SEER MP-SIR.

Supplementary Table 9. Potential site SIRs sub-grouped by different attained age of index cervical cancer survivors, SEER, 1988–2015, with 12 months latency since the initial primary diagnosis.

Site of second primary malignancy	20–39		40–59		60–79		≥80	
	O/E	SIR (95% Cl)#	O/E	SIR (95% Cl)#	O/E	SIR (95% Cl)#	O/E	SIR (95% Cl)#
HPV-related SPMs	6/1	5.5* (2.0–12.0)	69/20	3.5* (2.8–4.5)	60/21	2.8* (2.1–3.6)	16/6	2.8* (1.6–4.5)
Oropharynx	0/0	0	7/3	2.1 (0.8–4.3)	4/4	1.1 (0.3–2.9)	0/1	0
Female Genital System	5/0	13.9* (4.5–32.3)	44/5	9.4* (6.8–12.7)	39/6	6.8* (4.8–9.4)	11/2	5.7* (2.7–10.5)
Vagina	5/0	106.4* (34.6–248.4)	28/1	37.9* (25.2–54.8)	19/1	19.4* (11.7–30.2)	5/0	15.8* (5.1–37.0)
Vulva	0/0	0	14/3	4.7* (2.4–8.2)	17/3	5.2* (2.9–8.5)	5/2	3.5 (1.0–8.9)
Anus, Anal Canal and Anorectum	1/0	11.5 (0.3–64.0)	4/3	1.5 (0.4–3.7)	5/2	2.1 (0.7–5.0)	0/0	0
Rectum	0/0	0	14/8	1.8* (1.0–2.9)	12/10	1.3 (0.7–2.3)	5/2	2.1 (0.8–4.5)
Smoking-related SPMs	7/1	7.1* (2.9–14.6)	142/43	3.3* (2.8–3.9)	223/109	2.0* (1.8–2.3)	48/27	1.8* (1.3–2.3)
Esophagus	0/0	0	3/1	2.0 (0.4–5.9)	7/4	1.8 (0.7–4.0)	0/1	0
Lung and Bronchus	5/1	6.8* (2.2–16.0)	123/36	3.4* (2.8–4.1)	180/91	2.0* (1.7–2.3)	39/20	1.9* (1.3–2.6)
Bladder	2/0	8.7* (1.1–31.6)	16/6	2.8* (1.5–4.6)	36/14	2.5* (1.7–3.4)	9/5	1.7 (0.8–3.2)
Hormone-related SPMs	22/14	1.5 (0.9–2.3)	169/210	0.80* (0.68–0.93)	123/190	0.65* (0.54–0.77)	25/37	0.70 (0.44–1.05)
Female Breast	20/13	1.5 (0.9–2.3)	150/193	0.77* (0.65–0.90)	112/174	0.64* (0.53–0.78)	18/33	0.55* (0.31–0.89)
Ovary	2/1	1.7 (0.2–6.2)	19/17	1.2 (0.7–1.9)	11/17	0.66 (0.33–1.17)	7/4	1.9 (0.8–3.9)

Abbreviations: SIR: standardized incidence ratio; SPMs: second primary malignancies; SEER: surveillance, epidemiology, and end results; CI: confidence interval; O: observed number of cancer patients; E: expected number of cancer patients; SPMs: second primary malignancies; HPV: human papillomavirus. *P value of SIR <0.05 was considered significant. #The 95% CI was automatically generated by the SEER MP-SIR.

Supplementary Table 10. Potential site SIRs sub-grouped by different attained age of index endometrial cancer survivors, SEER, 1988–2015, with 12 months latency since the initial primary diagnosis.

Site of second primary malignancy	20–39		40–59		60–79		≥80	
	O/E	SIR (95% Cl)#	O/E	SIR (95% Cl)#	O/E	SIR (95% Cl)#	O/E	SIR (95% Cl)#
HPV-related SPMs	3/0	10.4* (2.1–30.4)	44/32	1.4 (1.0–1.8)	176/144	1.2* (1.1–1.4)	76/65	1.2 (0.9–1.5)
Oropharynx	0/0	0	4/6	0.70 (0.19–1.78)	19/25	0.76 (0.46–1.19)	6/8	0.79 (0.29–1.71)
Female Genital System	3/0	35.0* (7.2–102.2)	18/7	2.5* (1.5–4.0)	76/40	1.9* (1.5–2.4)	32/21	1.5* (1.0–2.1)
Vagina	2/0	175.8* (21.3–635.0)	11/1	9.3* (4.7–16.7)	45/7	6.7* (4.9–9.1)	12/3	3.5* (1.8–6.1)
Vulva	1/0	21.2 (0.5–118.0)	5/4	1.3 (0.4–3.0)	31/22	1.4 (1.0–2.0)	19/15	1.3 (0.8–2.1)
Anus, Anal Canal and Anorectum	0/0	0	4/5	0.88 (0.24–2.26)	5/17	0.30* (0.10–0.70)	3/6	0.51 (0.10–1.48)
Rectum	0/0	0	18/15	1.2 (0.7–2.0)	76/62	1.2 (1.0–1.5)	34/30	1.2 (0.8–1.6)
Smoking-related SPMs	1/0	4.6 (0.1–25.4)	102/75	1.4* (1.1–1.7)	565/780	0.73* (0.68–0.80)	260/305	0.86* (0.76–0.97)
Esophagus	0/0	0	1/2	0.41 (0.01–2.29	17/22	0.83 (0.49–1.32)	10/11	0.90 (0.43–1.65)
Lung and Bronchus	1/0	6.1 (0.2–34.0)	82/64	1.3* (1.1–1.7)	442/651	0.68* (0.62–0.75)	183/233	0.79* (0.68–0.91)
Bladder	0/0	0	19/10	2.0* (1.2–3.0)	106/105	1.0 (0.8–1.2)	67/61	1.1 (0.9–1.4)
Hormone-related SPMs	8/4	2.2 (1.0–4.4)	265/318	0.84* (0.74–0.94)	1237/1271	0.98 (0.93–1.03)	385/365	1.1 (1.0–1.2)
Female Breast	3/3	0.90 (0.19–2.63)	243/292	0.84* (0.74–0.95)	1198/1157	1.0 (1.0–1.1)	377/321	1.2* (1.1–1.3)
Ovary	5/0	17.9* (5.8–41.7)	22/27	0.81 (0.51–1.23)	39/115	0.34* (0.24–0.46)	8/43	0.19* (0.08–0.37)

Abbreviations: SIR: standardized incidence ratio; SPMs: second primary malignancies; SEER: surveillance, epidemiology, and end results; CI: confidence interval; O: observed number of cancer patients; E: expected number of cancer patients; SPMs: second primary malignancies; HPV: human papillomavirus. *P value of SIR <0.05 was considered significant. #The 95% CI was automatically generated by the SEER MP-SIR.

Supplementary Table 11. Potential site SIRs by radiotherapy status of index cervical cancer and endometrial cancer survivors, SEER, 1988–2015, with 12 months latency since the initial primary diagnosis.

Site of second primary malignancy †	Cervical cancer, RT		Endometrial cancer, RT		Cervical cancer, no RT		Endometrial cancer, no RT	
	O/E	SIR (95% CI)#	O/E	SIR (95% CI)#	O/E	SIR (95% CI)#	O/E	SIR (95% CI)#
Lip	2/0	4.9 (0.6–17.6)	3/2	1.7 (0.3–4.9)	0/1	0	0/5	0
Salivary Gland	1/1	0.91 (1.02–5.05)	0/4	0	2/2	1.2 (0.2–4.4)	11/10	1.1 (0.6–2.0)
Floor of Mouth	2/1	3.8 (1.5–13.5)	2/2	1.2 (0.2–4.3)	1/1	1.4 (0.0–8.0)	1/4	0.22 (0.01–1.24)
Gum and Other Mouth	1/2	0.57 (2.01–3.2)	3/7	0.45 (0.09–1.31)	5/2	2.4 (0.8–5.5)	9/18	0.51* (0.23–0.96)
Nasopharynx	1/0	2.2 (0.1–12.2)	0/1	0	0/1	0	4/3	1.4 (0.4–3.7)
Hypopharynx	2/0	5.1 (0.6–18.4)	1/1	0.83 (0.02–4.61)	1/0	2.1 (0.1–11.6)	0/3	0
Other Oral Cavity and Pharynx	0/0	0	1/1	1.8 (0.0–9.7)	0/0	0	0/2	0
Stomach	11/8	1.4 (8.7–2.6)	20/22	0.9 (0.55–1.39)	7/8	0.84 (0.34–1.73)	43/59	0.73* (0.53–0.98)
Small Intestine	7/2	2.9* (2.2–6.0)	12/8	1.5 (0.8–2.7)	1/3	0.31 (0.01–1.75)	23/21	1.1 (0.7–1.6)
Cecum	16/10	1.6 (10.9–2.6)	47/39	1.2 (0.9–1.6)	9/10	0.87 (0.4–1.65)	109/99	1.1 (0.9–1.3)
Appendix	1/1	1.2 (1.0–6.8)	0/2	0	0/1	0	5/7	0.74 (0.24–1.73)
Ascending Colon	10/8	1.3 (8.6–2.4)	57/31	1.9* (1.4–2.4)	3/8	0.37 (0.08–1.07)	94/79	1.2 (1.0–1.5)
Hepatic Flexure	4/2	1.9 (2.5–5.0)	13/8	1.6 (0.9–2.8)	2/2	0.94 (0.11–3.38)	33/20	1.6* (1.1–2.3)
Transverse Colon	5/4	1.3 (4.4–3.1)	23/15	1.5 (1.0–2.3)	3/4	0.73 (0.15–2.14)	56/38	1.5* (1.1–1.9)
Splenic Flexure	1/1	0.79 (1.02–4.38)	5/4	1.2 (0.4–2.9)	2/1	1.4 (0.2–5.1)	12/11	1.1 (0.6–2.0)
Descending Colon	3/2	1.3 (2.3–3.8)	10/7	1.4 (0.7–2.6)	6/3	2.3 (0.8–4.9)	15/18	0.82 (0.46–1.35)
Sigmoid Colon	14/10	1.3 (10.7–2.3)	53/32	1.7* (1.3–2.2)	16/13	1.3 (0.7–2.1)	113/84	1.3* (1.1–1.6)
Large Intestine, NOS	6/2	2.6 (3.0–5.7)	12/9	1.3 (0.7–2.3)	1/2	0.43 (0.01–2.42)	15/24	0.63 (0.35–1.04)
Rectosigmoid Junction	8/4	2.1 (4.9–4.1)	22/11	1.9* (1.2–2.9)	3/5	0.63 (0.13–1.85)	36/30	1.2 (0.8–1.6)
Liver	2/5	0.44 (5.05–1.59)	12/13	0.91 (0.47–1.58)	7/5	1.3 (0.5–2.7)	36/37	0.98 (0.69–1.36)
Gallbladder	2/2	1.0 (2.1–3.7)	6/7	0.88 (0.32–1.92)	3/2	1.4 (0.3–4.1)	13/18	0.73 (0.39–1.24)
Intrahepatic Bile Duct	1/1	0.92 (1.02–5.15)	4/4	1.0 (0.3–2.6)	0/1	0	11/11	1.0 (0.5–1.8)
Other Biliary	1/2	0.53 (2.01–2.94)	9/7	1.2 (0.6–2.4)	0/2	0	15/19	0.78 (0.43–1.28)
Pancreas	21/15	1.4 (15.9–2.1)	46/56	0.81 (0.6–1.09)	4/17	0.24* (0.07–0.61)	143/149	0.96 (0.81–1.13)
Retroperitoneum	0/0	0	1/1	0.77 (0.02–4.28)	2/1	3.0 (0.4–10.9)	2/4	0.55 (0.07–1.99)
Peritoneum, Omentum and Mesentery	2/1	1.6 (1.2–5.9)	3/5	0.61 (0.13–1.79)	2/2	1.3 (0.2–4.5)	28/13	2.1* (1.4–3.0)
Other Digestive Organs	3/1	4.2 (1.9–12.3)	5/3	1.8 (0.6–4.2)	2/1	2.4 (0.3–8.6)	7/7	0.94 (0.38–1.94)
Nose, Nasal Cavity and Middle Ear	0/1	0	4/2	1.9 (0.5–4.9)	1/1	1.2 (0.0–6.6)	5/6	0.88 (0.29–2.06)
Larynx	2/2	1.2 (2.1–4.1)	7/5	1.4 (0.6–2.9)	6/2	2.6 (1.0–5.7)	7/14	0.51 (0.21–1.05)
Pleura	0/0	0	1/0	9.2 (0.2–51.2)	0/0	0	0/0	0
Trachea	0/0	0	1/0	5.2 (0.1–29.2)	0/0	0	0/1	0
Mediastinum and Other Respiratory Organs	0/0	0	0/0	0	0/0	0	2/0	4.9 (0.6–17.6)
Bones and Joints	2/1	3.7 (1.4–13.3)	1/1	0.68 (0.02–3.78)	0/1	0	2/4	0.48 (0.06–1.74)
Soft Tissue including Heart	9/3	3.4* (3.5–6.4)	11/8	1.3 (0.7–2.4)	4/4	1.0 (0.3–2.6)	22/23	0.96 (0.6–1.45)
Melanoma of the Skin	11/16	0.68 (16.34–1.22)	52/54	0.97 (0.72–1.27)	16/31	0.51* (0.29–0.83)	136/153	0.89 (0.75–1.05)
Other Non-Epithelial Skin	1/2	0.64 (2.02–3.57)	9/6	1.4 (0.7–2.7)	2/2	0.89 (0.11–3.21)	21/17	1.2 (0.8–1.9)
Prostate	0/0	0	0/0	0	0/0	0	0/0	0
Testis	0/0	0	0/0	0	0/0	0	0/0	0
Penis	0/0	0	0/0	0	0/0	0	0/0	0
Other Male Genital Organs	0/0	0	0/0	0	0/0	0	0/0	0
Kidney	12/11	1.1 (11.6–1.9)	41/37	1.1 (0.8–1.5)	15/16	0.97 (0.54–1.59)	110/101	1.1 (0.9–1.3)
Renal Pelvis	1/1	1.1 (1.0–6.3)	4/4	1.0 (0.3–2.6)	1/1	1.1 (0.0–5.9)	15/10	1.5 (0.8–2.4)
Ureter	2/0	4.0 (0.5–14.6)	6/2	2.8* (1.0–6.0)	3/1	5.9* (1.2–17.1)	4/6	0.71 (0.19–1.81)
Other Urinary Organs	2/0	7.8 (0.9–28.1)	1/1	1.1 (0.0–6.0)	0/0	0	5/2	2.0 (0.7–4.7)

Eye and Orbit - Non-Melanoma	0/0	0	0/0	0	0/0	0	1/1	1.3 (0.0–7.3)
Eye and Orbit - Melanoma	0/1	0	4/2	1.8 (0.5–4.5)	1/1	1.0 (0.0–5.8)	4/6	0.64 (0.17–1.63)
Brain	5/5	1.1 (5.3–2.5)	21/16	1.3 (0.8–2.0)	4/7	0.55 (0.15–1.4)	23/44	0.53* (0.34–0.79)
Cranial Nerves Other Nervous System	1/0	3.1 (0.1–17.4)	0/1	0	0/1	0	4/2	1.8 (0.5–4.6)
Thyroid	13/14	0.93 (14.5–1.59)	41/29	1.4* (1.0–1.9)	19/30	0.64 (0.39–1)	99/91	1.1 (0.9–1.3)
Thymus	1/0	2.9 (0.1–16.1)	1/1	1.1 (0.0–6.1)	0/1	0	2/3	0.77 (0.09–2.78)
Adrenal Gland	0/0	0	2/1	3.5 (0.4–12.7)	0/0	0	0/2	0
Other Endocrine	0/0	0	0/0	0	0/0	0	0/1	0
Mesothelioma	1/0	2.1 (0.1–11.7)	1/2	0.50 (0.01–2.8)	2/1	3.3 (0.4–12.0)	4/5	0.76 (0.21–1.95)
Kaposi Sarcoma	0/0	0	0	0	1/0	6.8 (0.2–37.7)	2/1	1.8 (0.2–6.6)

Abbreviations: SIR: standardized incidence ratio; SEER: surveillance, epidemiology, and end results; RT: radiotherapy; no RT: not received radiotherapy; CI: confidence interval; O: observed number of cancer patients; E: expected number of cancer patients. [*]*P* value of SIR <0.05 was considered significant. [#]The 95% CI was automatically generated by the SEER MP-SIR. [†]Cancers site SIR of the tongue, tonsil, oropharynx, vagina, vulva, anus, rectum, esophagus, lung and bronchus, bladder, female breast, and ovary were showed in manuscript.
